# Assessment of the relationship between genotypic status of a DT-diaphorase point mutation and enzymatic activity

**DOI:** 10.1054/bjoc.2000.1359

**Published:** 2000-10

**Authors:** V Misra, A Grondin, H J Klamut, A M Rauth

**Affiliations:** Department of Medical Biophysics, University of Toronto and Division of Experimental Therapeutics, Ontario Cancer Institute, 610 University Avenue, Toronto, Ontario, M5G 2M9, Canada

**Keywords:** DT-diaphorase, polymorphism, RT-PCR-RFLP, genotype, activity

## Abstract

DT-diaphorase, a cytosolic reductase, has been implicated as an activator of chemotherapeutic prodrugs and a detoxifier of certain potentially carcinogenic xenobiotics. A common C to T nucleotide 609 substitution in DT-diaphorase cDNA has been associated with protein instability and reduced catalytic activity. The degree to which the allelic status of the substitution correlates with enzymatic activity was assessed in 45 normal human skin fibroblast strains using a PCR-RFLP assay. Included in this study was the 3437T strain, which is unique in that it is heterozygous for the polymorphism yet contains undetectable enzymatic activity. An allele-specific RT-PCR-RFLP technique attributed this phenomenon to exclusive DT-diaphorase mRNA expression from the variant allele. Overlap in activities was observed between individual strains homozygous for the wild-type allele and heterozygotes, but the former group displayed enzymatic activity that was on average 2-fold higher. Western blot analysis of the two strains in this panel that are homozygous for the variant allele revealed that they express relatively low amounts of DT-diaphorase protein, consistent with the role of the substitution in protein instability. This work confirms that genotypic status is a reliable initial estimate of DT-diaphorase activity. © 2000 Cancer Research Campaign


*British Journal of Cancer* (2000) **83**(8), 998–1002
© 2000 Cancer Research Campaign
doi: 10.1054/ bjoc.2000.1359, available online at http://www.idealibrary.com on IDEAL®

### Assessment of the relationship between genotypic status of a DT-diaphorase point mutation and enzymatic activity

**V Misra, A Grondin, HJ Klamut and AM Rauth**

Department of Medical Biophysics, University of Toronto and Division of Experimental Therapeutics, Ontario Cancer Institute, 610 University Avenue, Toronto, Ontario, Canada, M5G 2M9

**Summary** DT-diaphorase, a cytosolic reductase, has been implicated as an activator of chemotherapeutic prodrugs and a detoxifier of certain potentially carcinogenic xenobiotics. A common C to T nucleotide 609 substitution in DT-diaphorase cDNA has been associated with protein instability and reduced catalytic activity. The degree to which the allelic status of the substitution correlates with enzymatic activity was assessed in 45 normal human skin fibroblast strains using a PCR-RFLP assay. Included in this study was the 3437T strain, which is unique in that it is heterozygous for the polymorphism yet contains undetectable enzymatic activity. An allele-specific RT-PCR-RFLP technique attributed this phenomenon to exclusive DT-diaphorase mRNA expression from the variant allele. Overlap in activities was observed between individual strains homozygous for the wild-type allele and heterozygotes, but the former group displayed enzymatic activity that was on average 2-fold higher. Western blot analysis of the two strains in this panel that are homozygous for the variant allele revealed that they express relatively low amounts of DT-diaphorase protein, consistent with the role of the substitution in protein instability. This work confirms that genotypic status is a reliable initial estimate of DT-diaphorase activity. © 2000 Cancer Research Campaign

**Keywords**: DT-diaphorase; polymorphism; RT-PCR-RFLP; genotype; activity

DT-diaphorase (NAD(P)H:quinone oxidoreductase (NQO1), Enzyme Commission No. 1.6.99.2) is a homodimeric flavoenzyme that acts on a wide range of cytosolic substrates including quinones and their derivatives (Ernster and Navazio, 1987). DT-diaphorase is present in many tissues but is most abundant in liver (Jaiswal, 1994) and can also be elevated in colon, liver, and breast tumours relative to surrounding normal tissue (Schlager and Powis, 1990). The obligate two-electron reductase activity of DT-diaphorase is the basis for its postulated role as a defense mechanism against the carcinogenic effects of quinone xenobiotics, which are reduced to hydroquinone products by DT-diaphorase, thereby bypassing the formation of semiquinones and subsequent reactive oxygen intermediates (Ernster and Navazio, 1987). A DT-diaphorase gene knockout mouse exhibits increased quinone sensitivity, consistent with its role as a protective mechanism against xenobiotic toxicity (Radjendirane et al, 1998). A broad variety of structurally unrelated compounds have been shown to induce DT-diaphorase expression (Begleiter et al, 1997).

Many chemotherapeutic agents can be reductively activated by DT-diaphorase (Workman and Stratford, 1993), but one concern with the clinical use of these prodrugs is the variability in DT-diaphorase enzyme activities observed in different tissue types and tumours. It has also been suggested that the therapeutic index of drugs activated by DT-diaphorase can be increased through transcriptional activators such as 1,2-dithiole-3-thiones which can selectively induce this enzyme in tumour cells (Begleiter et al, 1997). DT-diaphorase and related nitroreductases are also being studied as prodrug activating enzymes in virus directed enzyme–prodrug therapies (Warrington et al, 1998).

Traver et al (1992) identified a C to T base change at position 609 of the DT-diaphorase mRNA which would confer a proline to serine substitution at amino acid 187 of the DT-diaphorase protein. This base change has been associated with low or absent DT-diaphorase activity (Kuehl et al, 1995; Traver et al, 1997), and transfection of eukaryotic cells with DT-diaphorase expression vectors containing either a C or T nucleotide at position 609 demonstrated that the proline to serine substitution results in decreased catalytic activity and protein levels (Misra et al, 1998). Furthermore, mutant protein purified from an *E. coli* expression system exhibited 2% of the enzymatic activity of wild-type DT-diaphorase (Traver et al, 1997). Allelic distribution of this common polymorphism is in accordance with the Hardy–Weinberg equilibrium with estimates of the frequency of homozygous carriers of the 609T allele ranging from 0.16–0.49 depending on ethnic background (Kelsey et al, 1997; Traver et al, 1997).

Marshall et al (1991) observed a relationship between DT-diaphorase activity and mitomycin sensitivity in a group of skin fibroblast strains donated by members of a cancer-prone family. However, in one heterozygotic cell strain, 3437T, DT-diaphorase activity was undetectable (Kuehl et al, 1995), which conflicted with previous reports showing that the allelic status of the nucleotide 609 polymorphism is a predictor for enzyme activity, with heterozygotes displaying activities intermediate to homozygous 609C (wild-type) and 609T (mutant) cell strains (Traver et al, 1992). DT-diaphorase mRNA was present in this cell strain in similar quantities to cells exhibiting high DT-diaphorase activities although the allelic origin of this mRNA was not determined. These results bring into question the reliability of predictions of

*Received 16 September 1999*
*Revised 3 April 2000*
*Accepted 11 May 2000*

*Correspondence to*: V Misra

DT-diaphorase activity on the basis of the genomic status of the nucleotide 609 polymorphism.

In the present report, a panel of normal human skin fibroblast cell strains were assessed for the allelic distribution of the nucleotide 609 polymorphism and the relationship between allelic status and DT-diaphorase activity. Also, the allelic origin(s) of DT-diaphorase mRNA expression was determined in 3437T cells, as well as seven selected strains from the skin fibroblast panel.

### MATERIALS AND METHODS

### Chemicals and reagents

All reagents used for the DT-diaphorase enzymatic assay were purchased from Sigma Chemical Co. (St. Louis, MO, USA). Materials used for genomic and RT-PCR, including AmpliTaq polymerase, were obtained from Roche Molecular Systems Inc. (Branchburg, NJ, USA). *H*inf I and proteinase K were purchased from Gibco-BRL (Burlington, ON, Canada) and Nonident P-40, gelatin, and Tween-20 were obtained from Sigma Chemical Co. DT-diaphorase monoclonal antibodies B771 and A180 were kindly supplied by Dr David Ross (University of Colorado Health Sciences Center, Denver CO, USA). Mouse anti-β-tubulin was obtained from Sigma. The protease inhibitor 'cocktail' (cat. no. 1697498) was purchased from Roche Bioscience (Palo Alto, CA, USA).

### Cell culture

Skin fibroblast cell strains used in this study were generously provided by Dr Peter Ray and Ms Nancy Cracknel of the Hospital for Sick Children, Toronto, Canada. 3437T cells were supplied by Dr Malcom Paterson (Cross Cancer Institute, University of Alberta, Edmonton, Alberta, Canada). All cells were grown in Alpha Minimal Essential Medium supplemented with 10% fetal bovine serum (Cansera, Rexdale, ON, Canada) and maintained in a humidified atmosphere containing 5% $CO_2$ at 37°C.

### Genomic PCR-*H*inf I RFLP assay

Cells were grown in 175 $cm^2$ tissue culture flasks (Nalge Nunc International, Denmark), harvested by scraping and centrifuged at 250 ***g***. The pellet was resuspended in 1 ml lysis buffer (50 mM KCl, 10 mM Tris-HCl (pH 8.3), 2.5 mM $MgCl_2$, 0.5 μg proteinase K, 0.1 mg $ml^{-1}$ gelatin, 0.45% Nonident P-40, and 0.45% Tween-20) and heated at 55°C for 1 h followed by 95°C for 10 min. To assess the status of the nucleotide 609 polymorphism, primers were used to amplify the entire coding region of exon 6 producing a 405 bp product containing nucleotide 609 as well as a native *H*inf I site, as described previously (Goldberg et al, 1998).

### DT-diaphorase assay

DT-diaphorase enzyme activity was measured using the substrate 2,6-dichlorophenolindophenol (DCPIP) as previously described (Misra et al, 1998).

### mRNA profile assessment of nucleotide 609 polymorphism

Total RNA was isolated from selected fibroblast strains using the RNeasy Mini Extraction Kit (Qiagen Inc., Santa Clarita, CA, USA). 5 μg of total RNA was reverse transcribed using the SuperScript II Preamplification Kit (Gibco) and a DT-diaphorase gene-specific primer 5′ TCC CAA CTG ACA ACC AGA TC 3′ (nt 841–860 of DT-diaphorase cDNA) according to the protocol of the manufacturer. 10% of the RT reaction was PCR amplified in a mixture (50 μl total volume) containing reaction buffer (50 mM KCl, 10 mM Tris-HCl (pH 8.3), and 0.001% (w/v) gelatin), 1.25 mM $MgCl_2$, 0.2 mM dNTP mix and 5 U AmpliTaq Polymerase with 100 ng each of DT-diaphorase gene-specific primer: forward 5′ GCC ATT CTG AAA GGC TGG TT 3′ (nt 381–400 DT-diaphorase cDNA); reverse 5′ CCA TCA CTT GGG CAA GTC CA 3′ (nt 821–840 DT-diaphorase cDNA).

The 460 bp fragment was amplified using a 'Touchdown' PCR protocol (Don et al, 1991) consisting of a 3 min denaturation at 94°C, two cycles of 1 min denaturation at 94°C, 1 min annealing at 60°C, and a 2 min polymerization at 72°C, 12 cycles in which the annealing temperature was decreased by 1°C until a touchdown temperature of 48°C was reached, an additional 17 cycles with the 48°C annealing temperature, and a final extension for 10 min at 72°C. The PCR product was digested with *H*inf I, fractionated on a 1.2% agarose gel, and visualized by ethidium bromide (10 μg $ml^{-1}$) staining. The wild-type (609C) DT-diaphorase allele generates a 377 bp *H*inf I fragment, whereas the variant allele (609T) produces two *H*inf I fragments of 226 and 151 bp. A constitutive 83 bp *H*inf I fragment is produced in addition to the ones derived from the wild-type and variant alleles but not observed under the these gel electrophoresis conditions and served as a positive cutting control.

### Western blot analysis

DT-diaphorase protein expression was verified in selected cell strains. Fibroblast strains were grown to a density of $5 \times 10^6$ cells in 175 $cm^2$ tissue culture flasks, harvested by scraping, pelleted at 250 ***g*** for 5 min. Pellets were resuspended in PBS containing protease inhibitor 'cocktail'. Cell extracts were prepared, protein concentrations were determined and Western blot analysis carried out was as previously described (Misra et al, 1998).

### RESULTS

### 609T nucleotide substitution correlates with reduced DT-diaphorase activity in a panel of 45 normal human fibroblast cell strains

The C to T base change in nucleotide 609 of DT-diaphorase cDNA, predicted to confer a proline to serine substitution in amino acid 187 of DT-diaphorase protein, has been shown to impair catalytic activity and may result in protein instability (Traver et al, 1997; Misra et al, 1998). The 609T nucleotide substitution produces a novel *H*inf I restriction site (RFLP) in the codon for amino acid 187, which can be used to screen genomic material for this variant allele (Goldberg et al, 1998). RFLP analysis of 45 human skin fibroblast cell strains, whose characteristics are shown in Table 1, resulted in the following allelic distribution of the nucleotide 609 polymorphism: 60% wild-type for both alleles (C/C fibroblasts); 35.5% heterozygous (C/T fibroblasts); and 4.5% homozygous for the variant allele (T/T fibroblasts). Thirty-one of the strains were assessed independently 2–3 times with complete consistency of results.

© 2000 Cancer Research Campaign 

**Table 1** Characteristics of skin fibroblast strains used for allelic status and DT-diaphorase activity determination

| | |
|---|---|
| Number of strains | 45† |
| Percent male donors | 55 |
| Percent female donors | 45 |
| Average donor age | 3 years (1 day–22 years) |
| Average strain passage number | 9 (6–14) |

† Allelic status of nt 609 polymorphism: 27 (60%) C/C, 16 (35.5%) C/T, 2 (4.5%) T/T

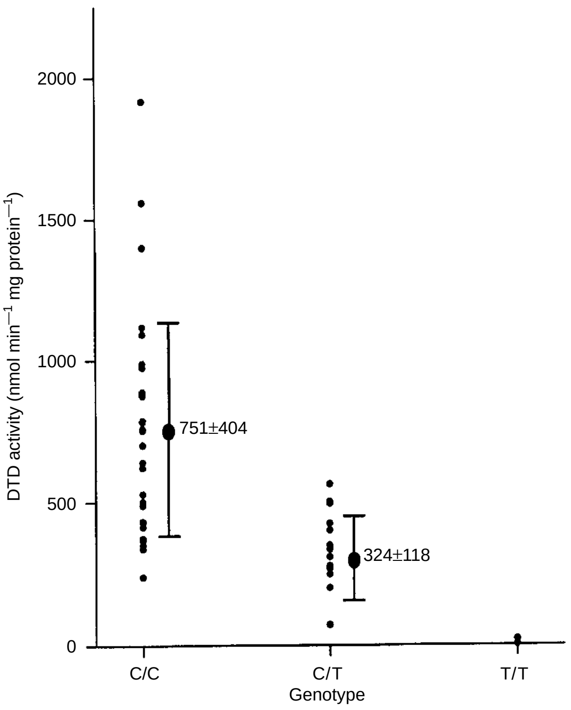

**Figure 1** Relationship between allelic status of nucleotide 609 polymorphism and DT-diaphorase activity. Each point represents an individual assessment of *H*inf I polymorphism status (allelic status of nucleotide 609 polymorphism) and DT-diaphorase activity for a single cell strain in a group of 45 normal human skin fibroblast strains. There was some overlap of data points. Mean ± SD DT-diaphorase activity of the group homozygous for the wild-type allele and the heterozygote group are indicated

An analysis of the relationship between allelic status and DT-diaphorase activity is shown in Figure 1. Each assay for enzymatic activity was run using three different concentrations of cell lysate and the resulting data was averaged to give a value which is plotted in the Figure. Repeat independent measurements with five of the cell strains which were C/C and three of which were C/T in genotype gave means with standard errors which were approximately plus or minus 20% of the mean ($n$ = 3–4). C/C and C/T fibroblasts displayed a wide range of DT-diaphorase activities, where the group homozygous for the wild-type (609C) DT-diaphorase allele had significantly greater enzymatic activities than heterozygous (609C/609T) cell strains (Mann–Whitney Rank Order Analysis, $\alpha < 0.01$ for a two-sided test) with the former showing 2-fold greater mean DT-diaphorase activities (754 ± SD 404 nmol min$^{-1}$ mg protein$^{-1}$) than the latter (324 ± SD 118 nmol min$^{-1}$ mg protein$^{-1}$). In two fibroblast strains that were homozygous for the variant allele, DT-diaphorase activity was extremely low to undetectable. These results are consistent with the notion that the 609T substitution impairs the catalytic activity and/or stability of DT-diaphorase, and that a correlation exists between 609T allelic status and DT-diaphorase activity in the individual cell strains studied.

### DT-diaphorase mRNA profile of 3437T fibroblasts

The 3437T cell strain is unusual in that it is heterozygous for the DT-diaphorase nucleotide 609 polymorphism but contains undetectable enzymatic activity (Marshall et al, 1991; Kuehl et al, 1995). These previous studies have shown that total DT-diaphorase mRNA levels in 3437T cells are similar to other cell strains displaying detectable enzymatic activity. Because cells that are homozygous for the variant allele display very low to undetectable DT-diaphorase activity, one possible explanation for the low DT-diaphorase activity in 3437T cells is that they express the variant 609T allele exclusively. To examine this question, the relative levels of expression of the two alleles in 3437T cells was compared to seven selected fibroblast strains from Table 1 that are homozygous for the wild-type allele (three strains), heterozygous (two strains) or homozygous for the variant allele (two strains). Total RNA isolated from each cell strain was PCR-amplified. As shown in Figure 2, the DT-diaphorase mRNA expression profile in the group of fibroblasts from Table 1 was in agreement with the genomic status of the nucleotide 609 polymorphism (with heterozygotes expressing DT-diaphorase mRNA from both alleles). However, 3437T cells displayed an RFLP pattern consistent with fibroblast strains that are homozygous for the variant (609T) allele, indicating that DT-diaphorase mRNA expressed in 3437T cells arises exclusively from the variant (609T) allele.

Because expression of the 609T variant allele has been associated with reduced DT-diaphorase protein levels (perhaps due to a decrease in protein stability), we also examined DT-diaphorase protein levels in 3437T cells relative to this same panel of seven fibroblast cell strains from Table 1. As shown in Figure 3, DT-diaphorase protein levels in 3437T cells were reduced relative to homozygous wild-type and heterozygous cell strains, to levels that were similar to that in the two fibroblast strains homozygous for the variant allele. This is consistent with the RT-PCR analysis pointing to exclusive expression of the variant 609T allele in 3437T cells. The enzymatic activity in all three of these cell strains was less than 20 nmol min$^{-1}$ mg protein$^{-1}$.

### DISCUSSION

The probability that cells will fail to activate bioreductive prodrugs or inactivate xenobiotics that are substrates for DT-diaphorase, due to expression of the 609T variant isoform, has been estimated by measuring the prevalence of the 609T allele in various populations. The frequency of the polymorphism varies between ethnic groups; with 2.5–25% of individuals homozygous for the variant 609T allele (Kelsey et al, 1997). The variant allele has also been examined in different tumour cell types, with reduced frequencies compared to disease-free reference groups observed in lung (Chen et al, 1999), and increased frequencies in certain infant leukaemias (Wiemels et al, 1999). However, studies showing no significant differences in variant allele frequencies in tumours have also been reported (Traver et al, 1997). A benzene-exposed population stratified into haematological malignancy and case control groups displayed a higher occurrence of the variant allele in the former group (Rothman et al, 1996), consistent with the possibility that

 *© 2000 Cancer Research Campaign*

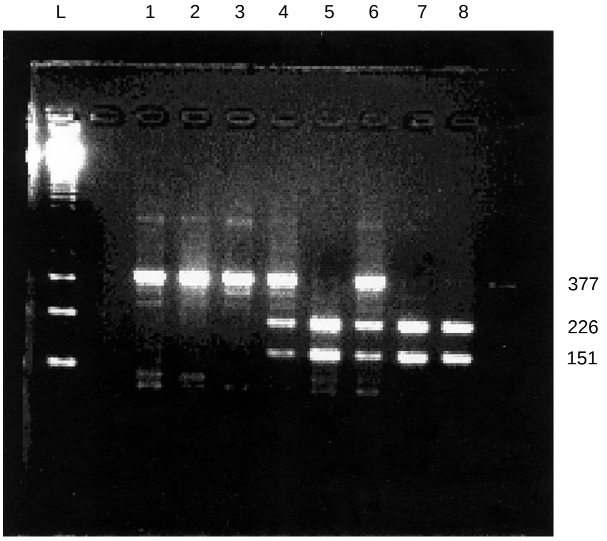

**Figure 2** DT-diaphorase allelic mRNA expression profiles. Seven skin fibroblast strains from Table 1 and 3437T cells were examined for wild-type and variant DT-diaphorase mRNA expression as described in Materials and Methods. The 377 bp band corresponds to wild-type allele expression while the 226 and 151 bp bands correspond to variant allele expression. Lanes 1–3, fibroblasts homozygous for the wild-type DT-diaphorase allele; Lanes 4 and 6, heterozygous fibroblasts; Lanes 7 and 8, fibroblasts homozygous for the variant allele; Lane 5, 3437T cells. L = 123 bp DNA size marker ladder

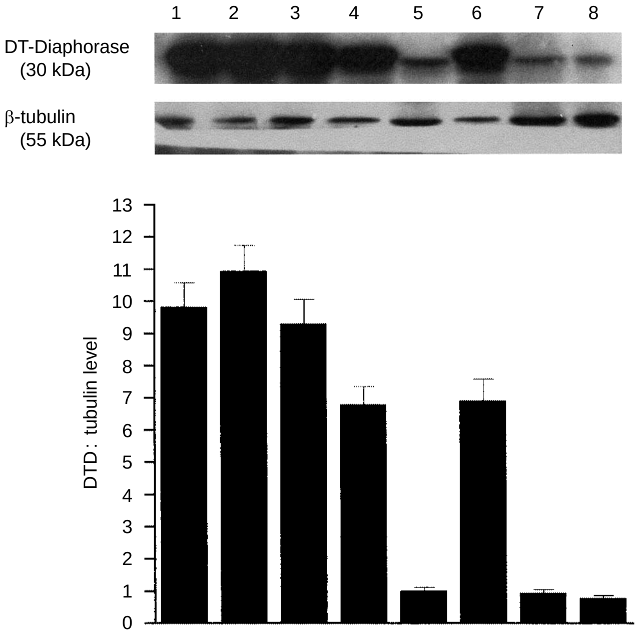

**Figure 3** Western blot analysis of DT-diaphorase protein levels in normal human skin fibroblast strains and 3437T cells. Immunoblot analysis was performed on total protein extracts using a mixture of DT-diaphorase monoclonal antibodies B771 and A180 as described in Misra et al (1998). Blots were also probed for β-tubulin expression as a protein-loading control. Lanes 1–3, normal human fibroblasts homozygous for the wild-type (609C) DT-diaphorase allele; Lanes 4 and 6, heterozygous fibroblasts; Lanes 7 and 8, fibroblasts homozygous for the variant allele (609T); Lane 5, 3437T cells. Lanes 5, 7, and 8 were intentionally loaded with 2-fold the protein as the other lanes. Also shown are DTD: β-tubulin expression for these strains as determined by densitometry. Values represent mean ± SD of five densitometric readings for each lane standardized to 3437T cells expression of the variant allele can impair the defense mechanism of the cell against carcinogenesis mediated by certain xenobiotics.

Estimating the magnitude of the response of individuals or cell populations to xenobiotics or prodrugs that are DT-diaphorase substrates on the basis of the allelic status of the nucleotide 609 polymorphism will depend on the extent to which allelic status reflects enzyme activity. The variant 609T allele has been implicated as a marker for some diseases associated with insufficient detoxification of xenobiotics by DT-diaphorase, and occurs at a higher frequency in some tumours. However, the absence of significant levels (Marshall et al, 1991; Kuehl et al, 1995) of DT-diaphorase activity in at least one human cell strain heterozygous for the variant allele, 3437T, brought into question the relationship between genotype and phenotype. In this study we have re-examined the prevalence of the DT-diaphorase nucleotide 609T substitution in the normal population using 45 normal fibroblast cell strains derived from skin biopsies (Table 1), and have used this information to investigate the correlation between genotypic status and DT-diaphorase activity.

The variant 609T allele occurred with a frequency of 0.21 in our panel of 45 normal human skin fibroblast cell strains, with a distribution that is in accordance with Hardy–Weinberg equilibrium and is consistent with previous reports (Kelsey et al, 1997). However, the aforementioned variation in frequency based on ethnicity may be relevant as a risk factor for disease associated with environmental and dietary exposures, as hypothesized for other drug metabolizing enzymes that display genetic polymorphisms (Nebert et al, 1999). The ethnicity of all the donors in the present skin fibroblast panel was not revealed. Limited information indicated that they represented a mixture of ethnic backgrounds.

Fibroblasts homozygous for the wild-type (609C) allele displayed on average 2-fold greater DT-diaphorase activities as compared to heterozygous fibroblast strains in this panel. This observation is consistent with a gene–dosage effect arising from expression of the single wild-type 609C allele in heterozygous strains. The two homozygous 609T fibroblast strains contained very low to undetectable enzyme activities in agreement with all reports of other groups. However, considerable overlap in enzyme activity was observed between the individual homozygous wild-type 609C and heterozygous fibroblast cell strains. A likely explanation for the broad range of enzyme activities within these two groups is a variation in the levels of DT-diaphorase mRNA expressed in each cell strain. Traver et al (1992) observed a strong correlation between DT-diaphorase mRNA levels and enzyme activity in a colon cancer cell panel using a semi-quantitative RT-PCR technique. Siegel et al (1999) also showed that quantification of DT-diaphorase protein levels in human saliva was directly correlated with the DT-diaphorase nucleotide 609 genotype with C/C individuals showing twice the protein level as C/T individuals and T/T individuals having undetectable levels. A similar overlap between C/C individuals and C/T individuals was seen in this work.

The 3437T cell strain in unique among cell strains in the present work and in previous studies, in that it is genetically heterozygous but displays undetectable DT-diaphorase activity. Western blot analysis indicates that these cells express DT-diaphorase at lower

© 2000 Cancer Research Campaign levels than cells homozygous for the wild-type allele, perhaps due to instability of the variant isoform. RT-PCR analysis confirmed that the variant allele is expressed exclusively in 3437T cells. Therefore, although 3437T cells represent an extreme case, they illustrate a potential problem in the use of allelic status of the nucleotide 609 polymorphism to predict DT-diaphorase activity in heterozygotes. The overlap in enzymatic activities observed between homozygous wild-type 609C and heterozygous fibroblasts also illustrates the difficulty in distinguishing C/C from C/T cells on the basis of enzyme activity, although such cell strains are clearly distinguishable from T/T cell strains.

The absence of measurable DT-diaphorase mRNA expression from the C allele in 3437T cells may be due to methylation leading to maternal or paternal gene silencing or to defects in the upstream promoter region effecting basal transcription. Similarly, the variation in DT-diaphorase activities observed within groups of C/C and C/T cells may reflect individual differences in transcriptional responses, given the complexity of pathways involving the xenobiotic response element and the aromatic response element (Favreau and Pickett, 1991) and their corresponding *cis*- and *trans*-acting factors.

In conclusion, accurate prediction of cellular responses to prodrugs and xenobiotics that depend on DT-diaphorase activity can be based to a first approximation on genotype status, which can distinguish between significant levels of enzyme activity in homozygous wild-type and heterozygous cells and very low to undetectable levels of enzyme activity in homozygous variant cells. Although heterozygotes have on average half the activity of wild-type cells, the ranges are overlapping. The existence of a heterozygous cell strain with no detectable DT-diaphorase activity, due to allele-specific expression, does not appear to be a common occurrence and thus will not play a large role in predictions based on genotyping.

### AKNOWLEDGEMENTS

This work was supported by the National Cancer Institute of Canada.

### REFERENCES

Begleiter A, Leith MK, Curphey TJ and Doherty GP (1997) Induction of DT-diaphorase in cancer chemoprevention and chemotherapy. *Oncol Res* **9**: 371–382

Chen H, Lum A, Seifried A, Wilken LR and Le Marchand L (1999) Association of the NAD(P)H:quinone oxidoreductase 609C to T polymorphism with a decreased lung cancer risk. *Cancer Res* **59**: 3045–3048

Don RH, Cox PT, Wainwright BJ, Baker K and Mattick JS (1991) 'Touchdown' PCR to circumvent spurious priming during gene amplification. *Nucleic Acids Res* **19**: 4008

Ernster L and Navazio F (1987) DT-diaphorase: A historical review. *Chemica Scripta* **27A**: 1–13

Favreau LV and Pickett CB (1991) Transcriptional regulation of the rat NAD(P)H: quinone oxidoreductase gene. Identification of regulatory elements controlling basal level expression and inducible expression by planar aromatic compounds and phenolic antioxidants. *J Biol Chem* **266**: 4556–4561

Goldberg ZL, Cummings BJ, Chapman WB, Klamut HJ and Rauth AM (1998) Role of a DT-diaphorase mutation in the response of anal canal carcinoma to radiation, 5-fluorouracil and mitomycin C. *Int J Radiat Oncol Biol Phys* **42**: 331–334

Jaiswal AK (1994) Human NAD(P)H:quinone oxidoreductase 2. Gene structure, activity, and tissue-specific expression. *J Biol Chem* **269**: 14502–14508

Kelsey KT, Ross D, Traver RD, Christiani DC, Zuo ZF, Spitz MR, Wang M, Xu X, Lee BK, Schwartz BS and Wiencke JK (1997) Ethnic variation in the prevalence of a common NAD(P)H quinone oxidoreductase polymorphism and its implications for anti-cancer chemotherapy. *Br J Cancer* **76**: 852–854

Kuehl BL, Paterson JW, Peacock JW, Paterson MC and Rauth AM (1995) Presence of a heterozygous substitution and its relationship to DT-diaphorase activity. *Br J Cancer* **72**: 555–561

Marshall RS, Paterson MC and Rauth AM (1991) DT-diaphorase activity and mitomycin C sensitivity in non-transformed cell strains derived from members of a cancer-prone family. *Carcinogenesis* **12**: 1175–1180

Misra V, Klamut HJ and Rauth AM (1998) Transfection of COS-1 cells with DT-diaphorase cDNA: role of a base change at position 609. *Br J Cancer* **77**: 1236–1240

Nebert DW, Ingelman-Sundberg M and Daly AK (1999) Genetic epidemiology of environmental toxicity and cancer susceptibility: human allelic polymorphisms in drug-metabolizing enzyme genes, their functional importance, and nomenclature issues. *Drug Metab Rev* **2**: 467–487

Radjendirane V, Joseph P, Lee YH, Kimura S, Klein-Szanto AJ, Gonzalez FJ and Jaiswal AK (1998) Disruption of the DT diaphorase (NQO1) gene in mice leads to increased menadione toxicity. *J Biol Chem* **273**: 7382–7389

Rothman N, Traver RD, Smith MT, Hayes RB, Li G-L, Campleman S, Dosemeci M, Zhang L, Linet M, Wacholder S, Yin S-N and Ross D (1996) Lack of NAD(P)H:quinone oxidoreductase activity (NQO1) is associated with increased risk of benzene hematotoxicity. *Proc Am Assoc Cancer Res* **37**: 258

Schlager JJ and Powis G (1990) Cytosolic NAD(P)H: (quinone-acceptor)oxidoreductase in human normal and tumor tissue: effects of cigarette smoking and alcohol. *Int J Cancer* **45**: 403–409

Siegel D, McGuinness SM, Winski SL and Ross D (1999) Genotype-phenotype relationships in studies of a polymorphism in NAD(P)H:quinone oxidoreductase 1. *Pharmacogenetics* **9**: 113–121

Traver RD, Horikoshi T, Danenberg KD, Stadlbauer TH, Danenberg PV, Ross D and Gibson NW (1992) NAD(P)H:quinone oxidoreductase gene expression in human colon carcinoma cells: characterization of a mutation which modulates DT-diaphorase activity and mitomycin sensitivity. *Cancer Res* **52**: 797–802

Traver RD, Siegel D, Beall HD, Phillips RM, Gibson NW, Franklin WA and Ross D (1997) Characterization of a polymorphism in NAD(P)H:quinone oxidoreductase (DT-diaphorase). *Br J Cancer* **75**: 69–75

Warrington KH Jr, Teschendorf C, Cao L, Muzyczka N and Siemann DW (1998) Developing VDEPT for DT-diaphorase (NQO1) using an AAV vector plasmid. *Int J Radiat Oncol Biol Phys* **42**: 909–912

Wiemels JL, Pagnamenta A, Taylor GM, Eden OB, Alexander FE and Greaves MF (1999) A lack of a functional NAD(P)H:quinone oxidoreductase allele is selectively associated with pediatric leukemias that have MLL fusions. United Kingdom Childhood Cancer Study Investigators. *Cancer Res* **59**: 4095–4099

Workman P and Stratford IJ (1993) The experimental development of bioreductive drugs and their role in cancer therapy. *Cancer Metastasis Rev* **12**: 73–82

*© 2000 Cancer Research Campaign*
